# A Simple Technique to Prevent Screw Stripping During Hardware Removal Using Bone Wax

**DOI:** 10.7759/cureus.39683

**Published:** 2023-05-30

**Authors:** Ramy Samargandi, Louis-Romée Le Nail

**Affiliations:** 1 Department of Orthopedic Surgery, Faculty of Medicine, University of Jeddah, Jeddah, SAU; 2 Department of Orthopedics and Traumatology, Centre Hospitalier Régional Universitaire (CHRU) de Tours, Tours, FRA

**Keywords:** bone wax, hardware removal, implant removal, orthopedics, screw stripping, surgical technique

## Abstract

Hardware removal is a common procedure in orthopedics, but it can be challenging and time-consuming. Difficulties in screw removal may arise due to bone growth or cement covering the screw heads, leading to screw damage, and increased surgical time. In this article, we describe a simple and inexpensive technique utilizing bone wax to protect screw heads from bone growth or cement, facilitating future implant removal. The application of bone wax over screw heads acts as a barrier, preventing bone growth or cement from engaging with the screw heads.

## Introduction

Hardware removal is a common procedure in orthopedics and can be indicated for several situations, including hardware irritation, infection, hardware breakage, revision for nonunion or malunion, and patient demand [1-3]. In some situations, hardware removal can be challenging, leading to an increase in surgical time, complications, and costs [2,3]. Various reasons may lead to difficult screw removal, including a damaged screw head that may occur after the removal of bone growth over the screw head or when the screw head is covered by bone cement during the revision of hip arthroplasty. Furthermore, bone growth or cement over the screw head may prevent aligning the screwdriver correctly into the screw head; loosening the screw without a good grip of the screwdriver into the screw head may lead to stripping of the recess of the screw heads and complicate the further attempt. Therefore, cleaning the head of the screw from bone, soft tissue, or bone cement before engaging the screwdriver is primordial to prevent stripping of the recess, but it may be time-consuming and may prolong surgical time [1]. Various techniques have been described for difficult implant removal in the medical literature. Several of these techniques have been described for many scenarios, such as stripped screw heads, broken screw heads, and buried broken screws [4-8]. However, there is a lack of research regarding technical tips that can prevent difficult implant removal.

Herein, we describe a simple inexpensive method for protecting the head of screws from bone growth or bone cement in various surgical situations, which facilitates future implant removal using bone wax.

## Technical report

We use bone wax as a mechanical barrier to protect head screws from bone formation or cement in various situations, such as isolated lag screws or cancellous screws, screws in plates, and screws over acetabular cage before cementation, preventing cement engagement to the head of the screw.

**Figure 1:**
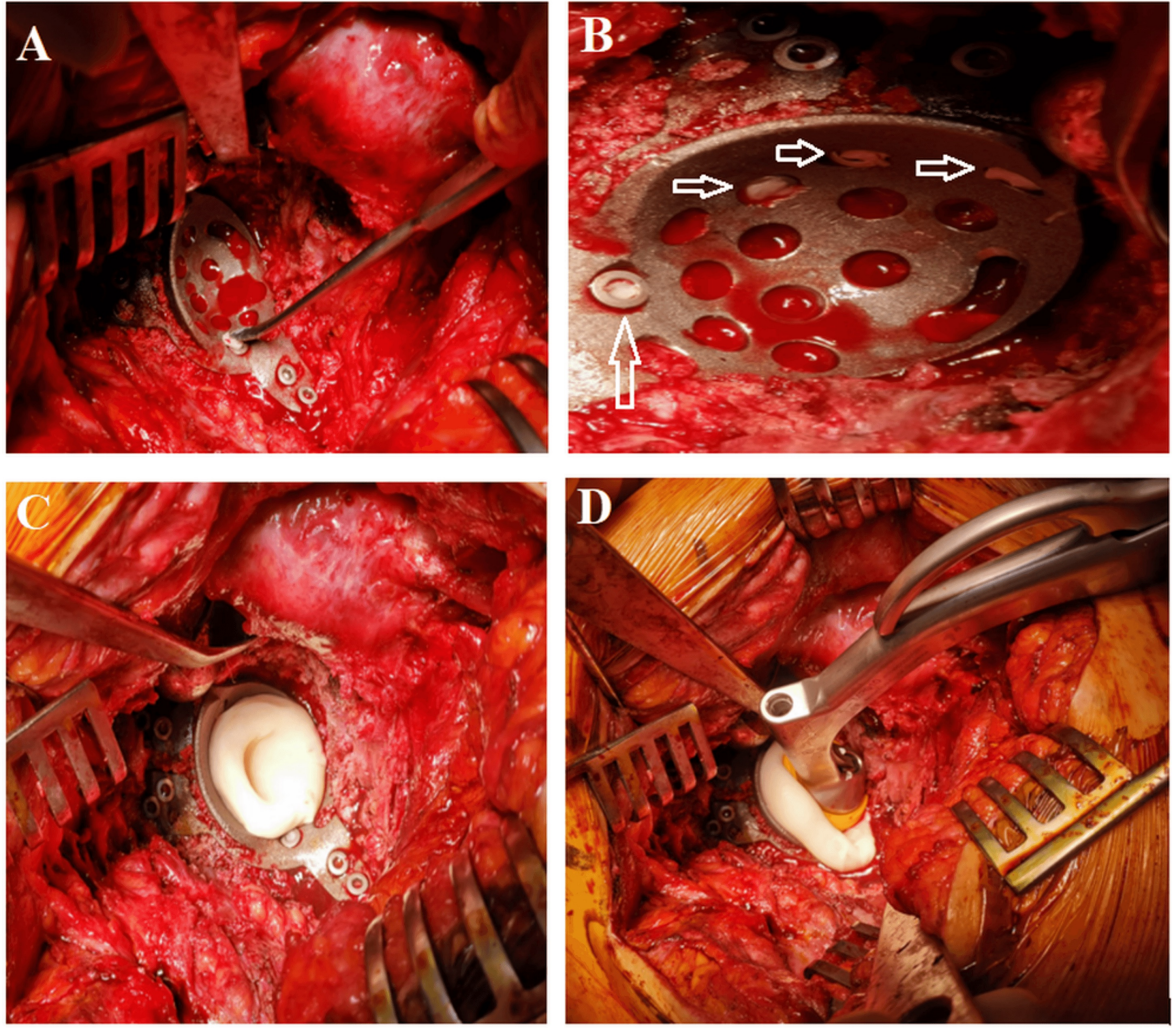
A: Bone wax is applied over the screw heads using a surgical spatula. B: Selective application of bone wax was performed on specific screw heads (arrows), while others were left without bone wax prior to cementation. C: Bone cement was applied over the screws. D: The cemented acetabular cup is positioned over the acetabular cage.

We apply a small quantity to cover the head of each screw before acetabular cup cementation in case the acetabular cage is used for total hip arthroplasty (Figure 1).

**Figure 2:**
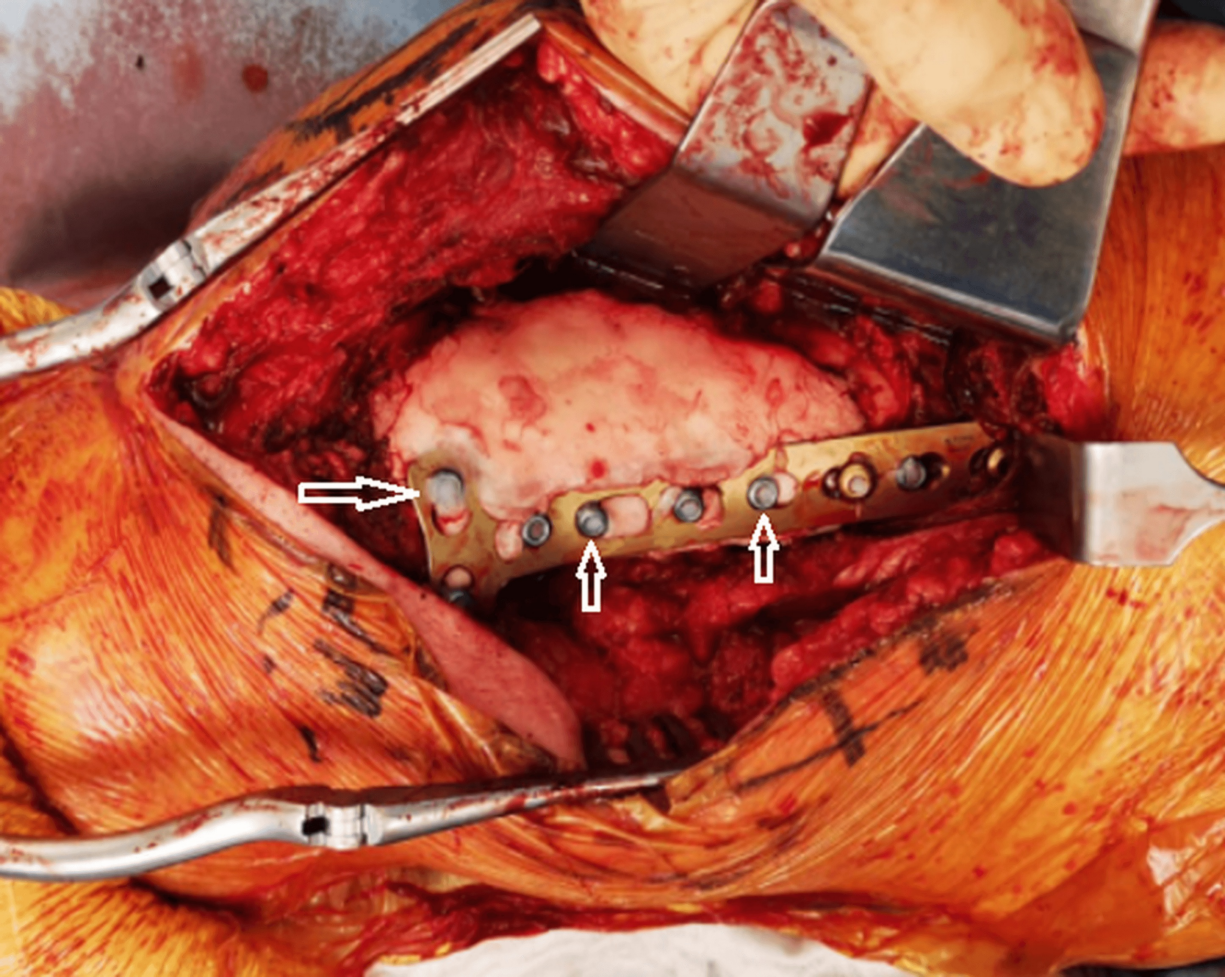
Following a hemicortical resection of bone metastasis from renal cell carcinoma and subsequent filling of the defect with bone cement in the distal femur, the plate was securely fixed in position. Prior to cementation, bone wax was carefully applied to the screw heads (arrows).

Additionally, bone wax is utilized to protect screw heads during cement use in tumor surgeries that involve curettage and filling with bone cement. It is also employed in the Masquelet technique for treating bone defects and in trauma cases following plate fixation (Figure 2).

This technique eases future implant removal and saves time during future implant removal by decreasing the time of removing bone growth or cement from the head of screws before screwdriver application, decreasing the risk of stripped head screws.

## Discussion

Bone wax is a nonabsorbable material that was first described by Henri Ferdinand Dolbeau in 1864 and then popularized by Sir Victor Horsley in 1892 [9,10]. Bone wax is a hemostatic agent commonly used in orthopedic surgery to control bleeding from bone surfaces, and it is an important tool in the armamentarium of the orthopedic surgeon [11]. It is composed of a mixture of beeswax and isopropyl palmitate or stearate, which forms a moldable and adhesive material. The use of bone wax has been prevalent in the field of orthopedics for over a century, owing to its effectiveness and safety. It is a simple and cost-effective tool that is readily available and easy to use. It can be applied to bone surfaces in a matter of seconds, making it a useful tool in emergency situations. Bone wax can also be removed easily.

Although bone wax is generally regarded as safe and has shown its advantages, it is important to be aware of potential complications that may arise from its use. One significant concern is the risk of infection [11-13]. As bone wax acts as a foreign body, it can create an environment conducive to bacterial colonization, potentially leading to postoperative infections. Although rare, bone wax can cause granulomas, characterized by chronic inflammation, which can also occur in some cases. These granulomas are thought to result from the body’s response to foreign material [14]. Furthermore, bone wax may interfere with bone healing. Some studies suggest that bone wax may impede the osteogenic process by inhibiting bone cell migration and proliferation [11,15,16]. It remains, however, debatable whether this effect is clinically significant and likely depends on the amount and location of bone wax used.

In our department, bone wax is commonly used to protect the heads of screws during revision total hip replacement surgeries when an acetabular cage is required as well as in tumors and trauma cases. This technique prevents cement from getting into the screw heads, making future removal easier. We have not encountered any complications reported in previous studies. Our hypothesis is that the lack of complications in our cases may be attributed to our specific technique of applying bone wax. In our technique, we only applied the bone wax to inert materials and in small amounts. Moreover, in hip arthroplasty cases, the bone wax was covered by cement, providing an extra layer of protection. This approach differs from previous reports where bone wax was directly applied to the bone surface [11-14]. We speculate that our modified application method may have played a role in the absence of complications.

## Conclusions

The application of bone wax as a protective barrier for screw heads in various orthopedic scenarios offers a simple and cost-effective method to facilitate future implant removal. By preventing bone growth or cement from engaging with the screw heads, this technique effectively reduces the risk of stripped heads and simplifies the removal process. Despite potential complications associated with bone wax, such as infection and foreign body reaction, the benefits of this technique outweigh the risks when used appropriately. Ultimately, the utilization of bone wax has the potential to save time, reduce surgical complexity, and assist orthopedic surgeons in facilitating future implant removal.
