# Correction: A Simple Technique to Prevent Screw Stripping During Hardware Removal Using Bone Wax

**DOI:** 10.7759/cureus.c289

**Published:** 2025-09-09

**Authors:** Ramy Samargandi, Louis-Romée Le Nail

**Affiliations:** 1 Department of Orthopedic Surgery, Faculty of Medicine, University of Jeddah, Jeddah, SAU; 2 Department of Orthopedics and Traumatology, Centre Hospitalier Régional Universitaire (CHRU) de Tours, Tours, FRA

The Ethics Statement and Conflict of Interest Disclosures section of this article has been corrected to indicate that this study did involve human subjects.

